# Does access to credit services influence availability of essential child medicines and licensing status among private medicine retail outlets in Uganda?

**DOI:** 10.1186/s40545-017-0116-8

**Published:** 2017-09-21

**Authors:** Lorraine Nabbanja Kabunga, Paschal Mujasi

**Affiliations:** 1 Universitat Pompeu Fabra, International Master in Health Economics & Pharmacoeconomics, Barcelona School of Management, Balmes 132, 08001 Barcelona, Spain; 2 International Masters in Health & Pharmacoeconomics; Universitat Pompeu Fabra, Barcelona School of Management, Balmes 132, 08001 Barcelona, Spain

## Abstract

**Background:**

Despite making great progress in reducing under five mortality in the last three decades. Uganda still ranks high among countries with the highest under five mortality rates. More than a third (36%) of these deaths are caused by pneumonia (15%), malaria (12%), or diarrhea (9%). For many mothers and caregivers, private drug shops are a point of care seeking for these illnesses. However, many drug-shops, are unlicensed and do not stock essential commodities due to insufficient capital and operational funds. This study set out to understand the relationship, between access to credit services through financial loans or stock and i) availability of essential child medicines and ii) licensing status among medicine retail outlet including drug shops and pharmacies.

**Methods:**

This was a cross-sectional study conducted between April and March 2016. The country was divided into 168 enumeration areas based on the geographical regions and household population distribution within the region; these served as the primary sampling units. Within each enumeration area, all private medicine retail outlets (drug-shops and pharmacies) that provide consultation for childhood illnesses were identified and surveyed. Data on access to credit services was collected through interviews and data on stock, through observations of shelves for Oral rehydration salts, amoxicillin dispersible tablets, amoxicillin syrup, Artemether combined therapies, and Zinc dispersible tablets. Android tablets were used for data collection and results were analyzed using STATA12. A total of 586 outlets were visited during the study, 96% were drug shops and 4% were pharmacies.

**Results:**

For all five essential child medicines assessed, access to credit through financial loans or through obtaining stock on credit did not influence stock availability. Access to credit services through loans or through stock on credit was seen to influence licensing status. The odds increased by more than 50% (1.53, CI: 1.27–2) among outlets who accessed loans compared to those who hadn’t and by 2 fold (2, CI: 1.03–3.8) among those who accessed stock on credit than in those who had not.

**Conclusions:**

Access to credit does not influence stock availability of essential child medicines among private medicine outlets, however, it has an effect on licensing status. In addition to further research, the provision of financing mechanisms to support the licensing processes could increase the proportion of unlicensed outlets.

## Background

Despite making great progress in reducing under-five mortality in the last three decades Uganda is still among countries in sub-Saharan Africa with high childhood mortality rates [1, 2]. Moreover, more than a third (36%) of these deaths are caused by three leading preventable and manageable childhood diseases; pneumonia (15%), malaria (12%), or diarrhea (9%) [3, 4].

More than 75% of caregivers in Uganda seek care outside of home for the mentioned illnesses [5]. About half of this proportion seeks care in the private sector, mainly in rural drug shops which account for over 80% of the private licensed outlets [6–8]. Drug shops in Uganda are outlets usually owned by medical personnel with at least nursing training. They are registered and licensed by Uganda National Drug Authority and allowed to sell nonprescription medicines [9]. Drug shops are a preferred point of care seeking due to, greater ease of access, shorter waiting periods, longer or more flexible opening hours, and better quality of services in general [10–15]. However, drug-shops have also been criticized for lacking valid permits, stocking medicines illegally and lacking appropriate standards of health care provision [16].

While access to affordable essential medicine has improved in the public sector the private sector continues to grapple with worrying trends [17]. Many private medicine retail outlets, particularly those in rural areas, do not stock essential commodities, including diagnostics such as malaria RDTs (available in just 13% of drug-shops and pharmacies) and respiratory rate counters for pneumonia (virtually absent at this level of the health care system) [18]. Preferred first line treatments such as ACTs for malaria, ORS and zinc for diarrhea are not regularly available; especially in pediatric formulations [19]. Quality-assured pediatric ACTs were available at just 4% of drug-shops and 45% of pharmacies [20].

Financial investment has been cited as a driver of stocking practices among private health care outlets. In Uganda access to credit, for example through microfinance services have shown improved availability of drugs at private clinics when using a client’s perspective [21]. Other studies have demonstrated, insufficient capital as a constraint among Accredited Drug Shops to stocking enough medicines [22].

Access to finance has been defined as the ability of individuals or enterprises to obtain financial services, including credit, deposit, payment, insurance, and other risk management services [23] Schreiner and Colombet define **microfinance** as “the attempt to improve access to small deposits and small loans for poor households neglected by banks” [24]. **Credit** on the other hand has been defined as a contractual agreement in which a borrower receives something of value now and agrees to repay the lender at some date in the future, generally with interest [25].

Promoting access to affordable financing or credit services for pharmaceutical private sector investments has been prioritized as a key intervention and deliverable for the Uganda Ministry of Health [26]. However, there is limited literature on access by private sector medicine retail outlets to credit either in form of loans from financing institutions or as stock of medicine from suppliers. Furthermore, it is unclear what association exists between access to credit and availability of essential child medicines among private medicine outlets or the availability of a license certificate.

### Rational and significance of study

There are currently no known studies that have measured the direct impact of credit on the availability of childhood medicines and licensing in Uganda specifically among first line private retail health service providers given their role as the first source of care for leading under five mortality diseases. Understanding the association between access to credit or loans and availability of essential child medicines will help determine whether financial capacity is indeed a barrier to stocking priority medicines. This could shape how interventions integrating finance and increasing essential child medicines availability in the private sector are designed.

### Aim and objectives

This study explores the level of access to credit services by private medicines outlets and the relationship between access to credit and: i) licensing status of medicine retail outlets as determined by availability of licensing permits; ii) availability of essential child medicines in private medicine retail outlets.

The specific study objectives were to:

- Determine the proportion of drug-shops and pharmacies receiving credit services for their operations
- Determine licensing status of the private medicine outlets in the study sample (drug shops and pharmacies)
- Determine the level of availability of five medicines used for diarrheal, pneumonia and malaria treatment for children under the age of 5 among different categories of private retail medicine outlets
- Explore the relationship between access to microfinance services and i) availability of diarrhea, pneumonia and malaria medicines for children under the age of five ii) Licensing status of the medicine retail outlet

Access to credit in this study was defined using two criteria; the ability to obtain loans by owners of private drug shops and pharmacies from financing institutions in the past one year or stock on credit from private suppliers (wholesalers or distributors) in the past 6 months.

### Hypothesis:

#### H1:

Private medicine retail outlets with access to credit show higher availability of essential child medicines and are more likely to be licensed compared to outlets without access to credit.

This hypothesis is based on the assumption that financial capacity either through loans or stock on credit is indeed a barrier to stocking essential child medicines and licensing among private medicine outlets.

## Methods

### Study design

This was a cross sectional study conducted by an independent organization between March and April 2016. The design was adopted to allow for investigation of a multiple outcomes at once and to be able to analyze correlations from the variables.

The study assessed several variables including stock availability of essential child health medicines among private medicine retail outlets, access to credit and licensing status. The essential child medicines assessed included Oral Rehydration Salts and dispersible Zinc tablets for diarrhea, Amoxicillin 125 mg syrups and dispersible tablets for pneumonia and Artemether Lumefantrine combined tablets for malaria treatments for children under 5.

### Sampling

The population of interest for the study was private sector medicine retail outlets in Uganda. The outlets included Pharmacies and drug shops. The respondents included attendants from the pharmacies and private drug shops. The study excluded traditional healers and individuals who sell or dispense medicine without providing any consultation for treatment.

The country was divided into 168 enumeration areas based on geographical regions and household population. Enumeration areas (EAs) served as the primary sampling unit and were used to determine the medicine retail outlets to be visited. An EA is a geographic area consisting of a convenient number of dwelling units that serve as counting units for the census.

To recruit outlets for the study, a two-stage cluster randomized sampling approach was used. Enumeration areas were stratified into regions and further into urban and rural areas, as defined by the 2002 census. EAs were then selected with probability proportional to size (PPS). Within each enumeration area, all private medicine retail outlets that provide consultation for childhood illnesses were identified and surveyed.

### Data collection and analysis

We used trained data collectors to visit and collect data from the selected private sector medicine outlets. The data collection involved a brief structured interview with the outlet attendant as well as an inspection of store inventory. For quality control, all data collectors were paired for each outlet visited. Only one data collector would ask the questions of the survey but both would record their responses. The data collectors assessed availability of the following five child medicines: Oral rehydration salts sachets, dispersible Zinc tablets, Artemether Lumefantrine oral tablets, Amoxicillin 125 mg syrups and Amoxycillin 125 mg tablets. Availability was defined as the presence of stock at the time of assessment as observed by the data collector. Licensing status was assessed based on the availability of a licensing certificates/permits issued by the National Drug Authority as observed by the data collectors.

Data was collected using Android tablets. Survey CTO software was used to script the paper versions onto the tablets Data was then extracted from the Survey CTO database and analyzed separately using STATA12. Univariate analyses were run to describe the characteristics of the outlets and bivariate analyses using odds ratios were run to determine the relationship between access to credit (the independent variable) and the two dependent variables; availability of essential child medicines and licensing status.

## Results

### Sample characteristics (univariate analysis)

A total of 586 outlets (drushops and pharmacies combined) were visited during the study, drug shops accounted for 96% while pharmacies 4%. The majority of pharmacies were located in urban settings (79%) while the majority of drugshops were placed in rural settings. Unlicensed outlets accounted for (71%). 90% of the outlets had been in operation for less than five years. There was an even split between outlets manned by owners versus outlets manned by employees at the time of the visit. These were the respondents in the survey. The majority of respondents were either nurses or midwives (82%). A much smaller proportion were part of a medical association (13%) (see Table 1).

**Table 1:** Outlet and respondent characteristics

	Type of outlet		
	Pharmacy n (%)	Drug shop n (%)	Total (%)
**Outlet characteristics**			
Number of outlets	24(4%)	562(96%)	586 (100%)
*Location*			
Urban	19(79%)	135(24%)	154(26%)
Rural	5(21%)	427(76%)	432(74%)
*Licensing Status*			
Licensed	20 (83%)	147(26%)	167(29%)
Not Licensed	4(17%)	409(74%)	413(71%)
*Period of operation*			
Less than five Years	15(79%)	369(81%)	384(90%)
Five and above Years	2(11%)	42(9%)	44(10%)
**Respondent characteristics**			
*Respondent interviewed*			
Outlet Owner	3(16%)	232(51%)	235(50%)
Outlet Employee	16(84%)	223(49%)	239(50%)
*Profession of respondent*			
Pharmacist	1(5%)	1(1%)	2(1%)
Pharmacy Technician	9(47%)	10(2%)	19(5%)
Medical Doctor	1(5%)	0(0%)	1(0%)
Nurse/midwife	6(32%)	279(71%)	285(82%)
Laboratory Technician	0(0%)	1(0.2%)	1(0.2%)
Clinical Officer	0(0%)	41(9%)	41(12%)
*Membership of Medical Association*			
Yes	4(21%)	53(12%)	57(13%)
No	11(58%)	355(78%)	366(87%)

### Funding and access to credit

#### Respondent’s knowledge about source of funding for the medicine outlet

In general, out of 586 medicine retail outlets (drug-shops and pharmacies combined), respondents from only 43%; 257 outlets (253 drugshops and 4 Pharmacies), were either knowledgeable of information on funds used to start or operate the outlets (including source). Of the 257 outlets, the majority was rural (78%; 201) and were drug-shops (98%; 253).

Of the knowledgeable proportion, respondents in only 47%; 122 outlets (121 drug-shops and 1 pharmacy) indicated that they knew about a potential source and could be able to obtain loans from financial credit institutions including banks, microfinance institutions or from Savings and Credit Cooperatives (SACCOs) within their communities if needed. The majority (77%) of respondents were in rural medicine outlets.

#### Source of funds for the medicine retail business start-up and operational costs

For the majority of outlets (67%; 172/257) respondents mentioned that personal savings of the owners were used to start the medicine retailer business.

A higher proportion of drug-shops (25%; 63/253) spent between 200,000–500,000 Uganda shillings (~60-150USD) on start-up costs. This was followed by drug-shops that spent between 500,000 to 1million Uganda shillings (~150-300USD) in startup costs at 23% (58/253).

Operational costs for a large proportion (34%; 87/257) of outlets ranged between 200,000 and 500,000 Uganda shillings (~60-150USD). Among pharmacies, the highest proportion of funds spent in startup costs were above 5million Ugx (~1500USD).

#### Access to credit in form of financial loans in the past one year

Out of 122 outlets whose attendants knew and could access funds, only 61 (50%) had actually obtained funds within the last one-year from a financial credit institution and all were drug-shops. None of the pharmacies had obtained funds from a financial credit institution. Some of the financial credit institutions mentioned included banks, local microfinance institutions, savings schemes and private lenders. For the majority of drug-shops, the amount of funds obtained from credit institutions ranged between 500,000 to 1million Uganda shillings. (~150-300USD).

**Fig. 1:**
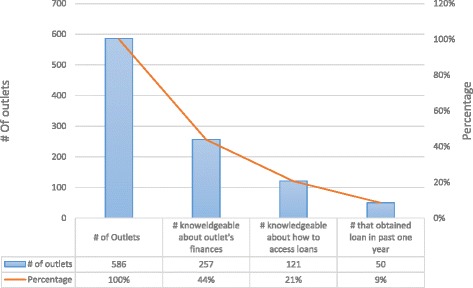
Knowledge about and access to credit among private medicine outlet

Figure 1 below describes the cascade of access to credit as loans from credit institutions among private medicine retailers. Overall, only 9% of the outlets in the study had accessed a financial loan in the past one year.

#### Access to credit in form of stock from supplier in the past six months

Among the 257 outlets whose respondents were knowledgeable about funding information of their outlets, only 32% (81) mentioned that the outlet had obtained credit in form of stock from their medicine suppliers. These included 77 drug-shops and all 4 pharmacies. Most outlets with access to stock credit from their medicine supplier were rural. The repayment period for the majority of drug-shops (40%) was 1 week compared to pharmacies for which the (75%; 3 out of 4) reported a 1 month repayment period from suppliers.

### Medicine availability

Pharmacies generally reported higher availability rates compared to drug shops for all five essential medicines assessed. 20% of the respondents refused to have availability measured during the assessment. Among drug shops, ACTs had the highest availability rates at 61% and Amoxicillin dispersible tablets had the lowest availability rates at 11%. Among pharmacies, Zinc dispersible tabs were the most widely available at 79% followed by ORS at 75%. Similarly to drug shops, Amoxicillin dispersible tablets were the least available among pharmacies at 38% (see Table 2).

**Table 2:** Stock availability of all five essential medicines

Product	Yes n (%)	No n (%)	Refused n (%)	Crude odds ratio (95% CI)	Adjusted odds ratio (95% CI)
Oral rehydration Salts					
Outlet type					
Drug shop	320(57)	132(23)	110(20)	1	1
Pharmacy	18(75)	1(4)	5(21)	7.4(.98–56.)	
Total	338(58)	133(23)	115(20)		
Zinc dispersible tablets					
Outlet type					
Drug shop	297(53)	155(28)	110(20)	1	1
Pharmacy	19(79)	0(0)	5(21)	1(0)	1(0)
Total	316(54)	155(26)	115(20)		
Artemether Lumefantrine combined (ACTs) oral treatments					
Outlet type					
Drug shop	344(61)	108(19)	110(20)	1	1
Pharmacy	17(71)	2(8)	5(21)	2.66(.60–11.7)	1
Total	361(62)	110(19)	115(20)		
Amoxicillin Dispersible tabs					
Outlet type					
Drug shop	61(11)	391(70)	110(20)	1	1
Pharmacy	9(38)	10(42)	5(21)	5.8(2.2–14.7)	
Total	70(12)	401(68)	115(20)		
Amoxicillin 125 mg syrups					
Outlet type					
Drug shop	270(48)	182(32)	110(20)		
Pharmacy	16(67)	3(13)	5(21)	3.5(1.03–12)	1
Total	286(49)	185(32)	115(20)		

### Relationship between access to credit and availability of child medicines (bivariate analysis)

Availability of the five child medicines for management of diarrhea, pneumonia and malaria varied between outlets with access to credit and those without access as shown in Table 3. 69% of the outlets that had accessed loans from credit institutions in the past one-year had ORS in stock at the time of the visit. A similar proportion (68%, 56 outlets) that did not access financial loans also had ORS in stock at the time of the visit. Similar rates of availability were observed among outlets that had accessed stock on credit from suppliers instead of financial loans from credit institutions.

**Table 3:** Relationship between Access to credit and medicine availability

Access to and type of credit	Stock			Odds ratios
	Yes n (%)	No n (%)	Crude odds ratio (95% CI)	Adjusted odds ratio^a^ (95% CI)
Oral Rehydration Salts (ORS)				
No Financial Loan	43(69)	19(31)	1	1
Financial Loan	40(67)	20(33)	1.03(.53–1.99)	1.12(0.56–2.23)
No Stock Credit	112(64)	64(36)	1	1
Stock Credit	60(74)	21(26)	1.07(0.65–1.720)	1.22(0.60–2.50)
Zinc dispersible Tablets				
No Financial Loan	41(66)	21(34)	1	1
Financial Loan	30(50)	30(50)	0.766(.40–1.43)	0.90(0.46–1.78)
No Stock Credit	104(59)	72(41)	1	1
Stock Credit	49(60)	32(40)	0.95(0.60–1.50)	1(0.50–2.0)
Artemether Lumefantrine combined oral treatments				
No Financial Loan	53(84)	9(16)	1	1
Financial Loan	42(70)	18(30)	0.46(.20–1.03)	0.45(0.18–1.1)
No Stock Credit	128(73)	48(27)	1	1
Stock Credit	60(74)	21(26)	1.19(0.70–2.00)	1.9(0.77–4.8)
Amoxicillin Dispersible tabs				
No Financial Loan	9(16)	53(84)	1	1
Financial Loan	9(15)	53(85)	1.14(0.5–2.52)	1.24(0.52–3.0)
No Stock Credit	27(15)	149(85)	1	1
Stock Credit	14(17)	67(83)	1.28(0.72–2.27)	1.16(0.50–2.7)
Amoxicillin 125 mg syrups				
No Financial Loan	50(81)	12(19)	1	1
Financial Loan	28(47)	32(53)	0.47(.24–0 .89)	0.43(0.23–0.9)
No Stock Credit	105(60)	71(40)	1	1
Stock Credit	54(67)	27(33)	1.16(0.73–1.84)	1(0.54–2.2)

^a^Adjusted for residence, outlet type and licensing status

More than half (58%) and slightly less than three thirds (61%) of outlets that had access to financial loans and stock on credit respectively had Zinc tablets available at the time of the visit. Higher availability of ACTs- (Artemether Lumefantrine Combined therapies/treatments) was observed among outlets that had no access to financial loans (87% of outlets) compared to outlets that had access (75% of outlets). A similar pattern was observed regarding availability of Amoxicillin syrup: 72% of outlets that did not have access to loans had Amoxicillin syrup available compared to 55% of outlets that had access to financial loans.

Outlets that had accessed stock on credit from suppliers generally had higher or similar availability rates among all five essential medicines. Outlets that had access to ORS stock on credit had higher availability of the product 74% compared to those that had not.

Overall, access to micro finance did not influence stock availability of the drugs as it can be shown in Table 3, all the confidence intervals of the odds ratios of stocking had a null value.

### Licensing among private medicine outlets

As mentioned in the section on background characteristics, only 29% (167/586) of all outlets combined had licenses. However this was driven by low availability among drug shops (26%) compared to pharmacies (83%).

### Relationship between access to credit and licensing

Only 37% and 52% of the outlets that had access to credit in form of financial loans and stock credit from suppliers respectively were licensed. The odds of being licensed increase by more than 50% among outlets who accessed financial loans compared to those who hadn’t and by 2-fold in those who accessed stock credit compared to those who did not, respectively when adjusted for location, (odds ratios and their 95% CI of 1.53 (1.27–2) and 2(1.03–3.8) respectively) (See Table 4).

**Table 4:** Relationship between Access to Credit and Licensure

	Licensing status		Odds ratios	
Access and type of credit	Licensed n. (%)	Unlicensed n. (%)	Crude odds ratio (95% CI)	Adjusted odds ratio (95% CI)
Financial Loan	31(37%)	53(63%)	1.50(1.27–2)	1.53 (1.27–2.0)^a^
No Financial Loan	44(54%)	38(46%)	1	1
Stock on Credit	63(52%)	56(48%)	2.4(1.5–3.8)	2(1.03–3.8)^a^
No Stock on Credit	71(32%)	153(68%)	1	1

^a^Significant at the 0.05 probability level

## Discussion

The study showed high availability rates for ACTs, ORS and Zinc DT which is a positive trend given the priority of these medicines in addressing under-five mortality. The study also showed that not all outlets could access financing institutions or stock on credit even with knowledge of where to access such services. This was driven by the high proportion of rural based respondents who likely had limited service providers within their communities compared to urban settings as literature has shown [27–29]. The limited access to financial loans could also be attributed to the challenges of accessing funds from formal financing institutions given that majority of outlets were small-scale businesses. Studies have shown that small size medium entrepreneurs like drug-shops are often not able to meet bank requirements for annual statements and business plans, Also, lending terms and conditions of financing institutions often prevent potential borrowers from seeking loans due to limited capacity to raise sufficient collateral and to make repayments as required [30–32]. Consequently, informal credit sources provide an easier alternative for most borrowers. Additionally, the proportion of outlets obtaining credit from suppliers in form of stock of medicines was higher compared to outlets that obtained loans from financing institutions further demonstrating the difficulty with accessing actual funds (paper work and loan processing time). In the grand scheme however, the overall proportion of outlets, which reported access to credit, was generally low which shows the need for such services for the private health sector in Uganda. Programs designed to improve this access would have to address the low awareness on operational and start up costs as demonstrated by this study. Furthermore, programs would have to manage the dynamics of rights to financial information, as the study showed the majority of respondents were not owners of outlets. Similar studies have shown the majority of employees are relatives of owners [33–35]. Outlet owners are more likely to know the financials of their business compared to their employees, however, employees may have better knowledge of operational costs.

Once general medicine availability and licensing status was established, we hypothesized that private medicine retail outlets with access to credit would show higher availability of essential child medicines and were more likely to be licensed compared to outlets without access to credit. However, the results did not support this hypothesis for stocking of essential medicines, as there was no observed statistically significant relationship between credit and availability for any of the five essential child medicines assessed. Instead, in the case of ORS, Zinc, ACTs, and Amoxicillin syrups, findings showed a reverse trend with slightly higher availability rates among outlets that had no access to credit through loans compared to outlets that had. These results contradict studies that have demonstrated perceived improved medicine availability as a result of improved credit capacity especially through financial loans. When private sector health workers in Uganda were given loans and business skills training, client perceived quality of care (including medicine availability) improved among clinics receiving the loans and business skills [36]. Clients were more likely to choose these clinics based on medicine availability, fair charges, cleanliness and confidentiality. However, our study assessed actual availability of essential medicines and was specific to treatments for diarrhea, pneumonia and malaria in contrast to the studies that used client perspectives as an outcome measure of availability.

This lack of influence of access to credit on medicine availability could also be attributed to a few more factors. First, lack of access to credit may not be a significant driver to stocking medicines for treatment of essential childhood illnesses. In general, there is limited literature on the impact of microfinance on health outcomes specifically on medicine availability. However, two randomized control trials showed that while credit initiatives e.g. microfinance had increased profitability of businesses of small-scale entrepreneurs, there was little impact of the businesses on health outcomes [37, 38]. The fact that similar availability rates were observed at the time of the visit for all the five medicines among outlets that had access to credit and among outlets that did not have, demonstrates alternative factors at play which influence stocking such as monitoring and supervision as has been shown in other studies [39].

Private medicine retail outlets have also been shown to have poor adherence to stocking recommended treatments due to weaker regulation and limited incentive to comply as studies have shown [40]. All the five medicines assessed were the recommended first line treatments for management of the three leading under five mortality diseases. Increasing financial capacity among retail outlets would not guarantee investment in stock adequacy without a strong regulatory structure, consequently the stocking trends would not be any different among outlets with access to credit and those without.

The study demonstrated a statistically significant relationship between access to credit and licensing status with the odds of being licensed increasing by more than 50% (OR: 1.53 CI: 1.27–2) among outlets which had accessed loans compared to those, which had not. The odds increased by 2-fold (OR: 2 CI: 1.03–3.8) among outlets that had accessed stock through credit from suppliers compared to those that had not. This is a key positive trend, which affirms the role of credit in incentivizing licensing of private medicine outlets as programs such as the Accredited Drug Seller initiative have shown [41]. The annual licensing fees for drug shops, levied by the Uganda National Drug Authority, ranges from 255,000–112,500 (77-34USD) for new applications depending on whether the outlet is based in the capital city or up-country [42]. This licensing fee is equivalent to about 25–50% of both start up and operational costs as demonstrated by this study. In fact, in some instances this licensing fee is equivalent to the outlet’s working capital. Anecdotal evidence has also shown additional hidden costs in supervisor fees of regulatory agents which further drive up the costs http://www.newvision.co.ug/new_vision/news/1332402/nda-doubles-license-fees-drug-shop-pharmacies. Given the magnitude and direct effect of the licensing fee on start up and operational costs, access to credit provides a clear incentive for licensure. This findings call for additional investment in operational research to test different mechanisms of integrating credit services into the licensing process. Possible strategies could include provision off incentive financial schemes, which allow private medicine retailers to pay for their licensures over a longer period of time as opposed to a onetime upfront payment.

This study also had some limitations. Information on access to credit was based on a self-reported response from the attendant and could not be verified resulting in response bias. This could have over or under estimated the actual proportion of outlets accessing credit. Secondly, the prioritization of essential child medicines as an indicator for medicine availability may not be representative for a large proportion of the outlets or other essential medicines since other factors such price may be at play. The selected essential child medicines may not be key cost drivers in operational costs of running the outlet. Furthermore, the sampling methodology resulted in higher proportion of rural retail outlets compared to urban, which could have also skewed the findings.

## Conclusion

The study showed high availability rates for ACTs, ORS and Zinc DT which is a positive trend given the priority of these medicines in addressing under-five mortality. However, there was no statistical significance observed between access to credit and availability of these medicines. These findings bring to question whether increasing access to credit services among private retail outlets can particularly increase availability of the Ministry of Health recommended treatments for diarrhea, malaria and pneumonia treatments for children under five years in Uganda. Further research is therefore needed to understand additional factors that influence stocking by product category of medicines to triangulate which commodities whose availability is driven by financial capacity and commodities, which show a trend similar to the findings of this study.

Given the statistical significance of the positive relationship between access to credit and licensing, interventions already demonstrating this strategy should be scaled and efforts to shape markets for microfinance in rural areas explored. From a practice perspective, the national drug authority should consider establishing partnerships with local microfinance institutions to run incentive schemes to improve licensing especially in rural settings.

## Abbreviations

ACTs: Artemether Lumefanctrine Combined therapies/treatments
ADS: Accredited Drugs Shops
CI: Confidence intervals
DT: Dispersible tabs
EAs: Enumeration Areas
IRB: Institutional Review Board
MOH: Ministry of Health
NDA: National Drug Authority
ORS: Oral Rehydration Salts
PPS: Probability Proportional to Size
RDTS: Rapid Diagnostic tests
SACCOs: Savings and Credit Cooperatives
TASO: The Aids Support Organization
USD: US dollars
